# Bather Shedding as a Source of Human Fecal Markers to a Recreational Beach

**DOI:** 10.3389/fmicb.2021.673190

**Published:** 2021-06-25

**Authors:** Dong Li, Laurie C. Van De Werfhorst, Brandon Steets, Jared Ervin, Jill L. S. Murray, Naresh Devarajan, Patricia A. Holden

**Affiliations:** ^1^Bren School of Environmental Science and Management, University of California, Santa Barbara, Santa Barbara, CA, United States; ^2^Geosyntec Consultants, Santa Barbara, CA, United States; ^3^Creeks Division, Department of Parks and Recreation, Santa Barbara, CA, United States

**Keywords:** HF183 bacteroides, bathers, outfall, diurnal sources, chronic

## Abstract

Microbial source tracking (MST) can identify and locate surf zone fecal indicator bacteria (FIB) sources. However, DNA-based fecal marker results may raise new questions, since FIB and DNA marker sources can differ. Here, during 2 years of summertime (dry season) MST for a Goleta, California recreational beach, surf zone FIB were mainly from gulls, yet low level human-associated DNA-based fecal marker (HF183) was detected in 25 and 14% of surf zone water samples, respectively. Watershed sources were hypothesized because dry weather creek waters had elevated FIB, and runoff-generating rain events mobilized human (and dog) fecal markers and *Salmonella* spp. into creeks, with human marker HF183 detected in 40 and 50% of creek water samples, dog markers detected in 70 and 50% of samples, and *Salmonella* spp. in 40 and 33.3% of samples, respectively over 2 years. However, the dry weather estuary outlet was bermed in the first study year; simultaneously, creek fecal markers and pathogens were lower or similar to surf zone results. Although the berm breached in the second year, surf zone fecal markers stayed low. Watershed sediments, intertidal beach sands, and nearshore sediments were devoid of HF183 and dog-associated DNA markers. Based on dye tests and groundwater sampling, beach sanitary sewers were not leaking; groundwater was also devoid of HF183. Offshore sources appeared unlikely, since FIB and fecal markers decreased along a spatial gradient from the surf zone toward nearshore and offshore ocean waters. Further, like other regional beaches, surf zone HF183 corresponded significantly to bather counts, especially in the afternoons when there were more swimmers. However, morning detections of surf zone HF183 when there were few swimmers raised the possibility that the wastewater treatment plant (WWTP) offshore outfall discharged HF183 overnight which transported to the surf zone. These findings support that there may be lowest achievable limits of surf zone HF183 owing to several chronic and permanent, perhaps diurnal, low concentration sources.

## Introduction

Fecal contamination of recreational coastal waters is a global public health concern. Worldwide there are annually more than 120 million gastrointestinal and 50 million respiratory illnesses estimated to be associated with activities in contaminated coastal waters (Shuval, 2003). To determine microbial water quality as related to public health, routine monitoring of fecal indicator bacteria (FIB) including total coliforms, fecal coliforms, *Escherichia coli*, and enterococci may be mandated, such as by Assembly Bill 411 (AB411) in California (Sikich et al., 2018), with swimming advisories and beach closures implemented accordingly. However, by epidemiological studies, FIB may not relate to human illness (Colford et al., 2007; Arnold et al., 2013), as FIB may originate from animal feces that are of low risk to human health due to host specificity of pathogens, particularly enteric viruses (Sinclair et al., 2009). Fecal contamination in recreational waters is in fact frequently attributed to gulls and canines, with management practices implemented and FIB successfully diminished (Wright et al., 2009; Converse et al., 2012; Goodwin et al., 2016). Further, FIB can survive and grow in diverse environments including soil, sediments, beach sand, wrack and aquatic vegetation (Field and Samadpour, 2007; Harwood et al., 2014). As such, microbial source tracking (MST) is needed to discern FIB sources that should be remedied for protecting public health.

Modern MST typically discovers FIB sources by analyzing field samples for molecular markers that target genes of host-coevolved or -associated bacteria, using quantitative PCR (qPCR) or droplet digital PCR (ddPCR) (Field and Samadpour, 2007; Boehm et al., 2013, 2015; Harwood et al., 2014; Mayer et al., 2018). Although host-associated microorganisms and related DNA markers decay in marine waters (Green et al., 2011; Ahmed et al., 2014; Mattioli et al., 2017), markers can sensitively and specifically reveal hosts’ wastes, and allow for quantitative microbial risk assessment (QMRA) (Boehm et al., 2015; Brown et al., 2017b). As such, host-associated fecal markers such as the human fecal marker HF183 targeting bacteria closely related to *Bacteroides dorei* have been widely applied in numerous MST studies (Bernhard and Field, 2000; Field and Samadpour, 2007; Boehm et al., 2013, 2015; Harwood et al., 2014; Mayer et al., 2018), with their use now mainstream as an EPA-validated method.

However, MST at urban or suburban beaches is complex, owing to numerous potential human fecal sources including sanitary sewers and storm drains (U.S. Environmental Protection Agency, 2004; Griffith et al., 2013; Cao et al., 2017). Leaking sewers and sewer overflows can occur in coastal areas (Sikich et al., 2018). Urban stormwater runoff can transport FIB and pathogens directly into coastal waters (U.S. Environmental Protection Agency, 2004). Watershed creeks can transport fecal contamination to beach waters, including through coastal lagoons or transitional waters (Ervin et al., 2014; Riedel et al., 2015). Discerning the numerous proximate and distal sources potentially contributing to surf zone fecal contamination therefore requires systematic sampling within study designs, as per MST guidelines in the California Microbial Source Identification Manual (Griffith et al., 2013). Additionally, bather shedding can contribute FIB and pathogens such as *Staphylococcus aureus* directly to marine water (Elmir et al., 2007, 2009; Plano et al., 2011), and thus acts as a non-point source of fecal contamination in recreational coastal waters.

Still, questions remain at many beaches, as evidenced by the low but chronic presence, with few actionable explanations, of human fecal markers along the California coastline (Santoro and Boehm, 2007; McQuaig et al., 2012; Russell et al., 2013; Riedel et al., 2015; Cao et al., 2017; Jennings et al., 2018). FIB surf zone exceedances can be rare in California (Sikich et al., 2018), yet surf zone HF183 markers may persist at low levels, even after management practices are implemented to address diverse potential fecal sources such as sewage spills, and contaminated drains, creeks, and rivers (Ervin et al., 2014; Goodwin et al., 2016; San Diego Regional Water Quality Control Board, 2017; Los Angeles Regional Water Quality Control Board, State of California, 2018; Sikich et al., 2018). Resolving lingering human marker-related questions after FIB sources are identified is needed to fully understand potential human health risks, and adopt additional management actions accordingly.

Here, a popular California recreational beach located in Goleta, CA with approximately 1.5 million visitors annually was studied to determine the origins of historically elevated surf zone water FIB^1^. A 2-year comprehensive MST investigation involved hypothesizing fecal sources, then sampling and analyzing FIB, several host fecal markers, and human pathogens (collectively abbreviated as “FMPs,” for FIB, fecal markers, and pathogens). All conceivable sources and processes were investigated including watersheds, sanitary sewers, groundwater, intertidal beach sands, nearshore sediments, a wastewater treatment plant (WWTP) offshore outfall, and natural regional background levels. Impacts of surf zone human recreation during holidays or high visitation weekends and at different times of the day were also evaluated for their relationship to surf zone microbial water quality. By examining all potential fecal contamination sources and transmission routes, the origins of FIB contamination to Goleta Beach (GB) were revealed, together with the probable sources of low human fecal markers. The results herein shed light on a longstanding question regarding chronic surf zone HF183, and contribute to broadly understanding apparent fecal contamination in recreational beaches when most obvious or actionable sources are ruled out.

## Materials and Methods

### Site Description, Sampling Locations, and Hypotheses

GB, located in Goleta, CA (latitude 34.4172186, and longitude −119.8295828) in the public GB Park, is popular for swimming and fishing, and near the University of California, Santa Barbara campus point, which is popular for surfing. The beach and its location were recently described (Li et al., 2020), owing to GB’s use as a disposal site for contaminated sediments following a regional debris flow that occurred after the MST study herein. Some results from the study herein were published to establish the 2017 background surf zone water quality for comparison to the study of 2018 sediment disposal effects (Li et al., 2020). However, the major findings of this study, and most data, are heretofore unpublished.

The watersheds upstream of GB, including Atascadero Creek, San Jose Creek, and San Pedro Creek, merge in an estuarine slough that discharges into the GB surf zone between sampling sites G01 and G02 when the beach berm is breached (Supplementary Figure 1). Historical FIB exceedances in GB surf zone waters and the upstream watershed waters including the slough were available prior to this study (see text footnote 1). Geographic information system (GIS) layers were compiled to display creek locations, and relevant infrastructure data for beach sanitary sewers and storm drains, septic systems, and hardscape (Supplementary Figure 2). Field reconnaissance at the beaches and into upstream watersheds revealed visible potential fecal sources including groups of shorebirds and wrack on the beach, dogs and wildlife, homeless encampments in the watersheds, sanitary sewer manholes, storm drains, and restrooms. Based on background information and field reconnaissance, the following potential summertime sources and routes of fecal contamination to the surf zone were investigated: gulls, dogs, watershed waters and sediments, horses along Atascadero creek, runoff-generating rainfall events, intertidal beach sands, infrastructure including bathrooms, sewer lines and pump stations at the beach, nearshore and offshore marine water, treated WWTP effluent discharging through an ocean outfall (Supplementary Figure 2), and nearshore marine sediments. Bather shedding on holiday or high visitation weekends and at different times of the day was also studied, with the numbers of bathers in the surf zone and people on the sand recorded at the time of sampling. Regional background levels of FMPs were determined in the surf zone and creek locations at a reference beach, Arroyo Hondo, which is a natural preserve with little human activity as described elsewhere (Li et al., 2021). Samples of water from the surf zones, watersheds, groundwaters, nearshore, offshore, and WWTP ocean outfall, as well as intertidal beach sands, sediments from the watersheds and nearshore were collected during the AB411 (mostly dry) season (April–October) during 2016–2017 for the analyses of FMPs (Supplementary Figures 1, 3). Dry weather was defined as < 0.1” of rainfall in the preceding 72 h. Rain events that occurred during the AB411 season were sampled if predicted to be ≥ 0.2”. The details of the samples and results are in Supplementary Tables 1–7.

### Dye Studies

Dye studies were performed to assess sanitary infrastructure integrity including sanitary sewer lines, laterals, and pressurized force mains as potential sources of fecal contamination, and to determine if any leaking sewers were impacting groundwater or surface waters through high velocity groundwater or preferential flow pathways. A non-toxic fluorescent dye (2.5% Rhodamine WT dye, Cole-Parmer, Vernon Hills) was flushed down toilets in the five bathrooms and added directly to the three recreational vehicle (RV) sewer connections and two sewage lift stations located at GB Park. A total of 116 groundwater, 84 surf zone, and 63 slough water samples were collected from 6 groundwater, 4 surf zone, and 3 slough sampling locations after the addition of dye for dye testing (Supplementary Figure 4). One sample from each groundwater well was also collected and analyzed for FMPs. The details of studied infrastructure, temporary groundwater monitoring wells, dye addition, water sampling, and dye analysis are in the SI Methods.

### Sample Collection, Physicochemical Characterization, and FIB Analyses

The water sampling procedures for the surf zone, watershed, groundwater, nearshore, offshore, and WWTP ocean outfall diffuser are described in the SI Methods. Dissolved oxygen, electrical conductivity, and temperature of water samples were recorded in the field using an HQ40d multiparameter meter (Hach, Loveland, OH). The sampling details for watershed sediments, intertidal sands, and nearshore sediments, and the analyses of particle grain sizes, moisture, and total organic content are described in the SI Methods. Total coliform (TC), *E. coli* (EC) and enterococci (ENT) in water and solid samples were quantified using the IDEXX Quanti-Tray/2000 method, following the manufacturer’s protocols (IDEXX Laboratories, Westbrook, MA), with details in the SI Methods. FIB exceedances were based on California single sample surf zone criteria for TC (10,000 MPN/100 ml), EC (400 MPN/100 ml), and ENT (104 MPN/100 ml) (Sikich et al., 2018).

### DNA Extraction, PCR, qPCR, and ddPCR

The approaches for water and solid sample processing and DNA extraction have been described elsewhere (Li et al., 2020), and are detailed in the SI Methods. Briefly, DNA was extracted from water samples using the DNeasy PowerWater Kit (Qiagen, Carol Stream, IL) or the RNeasy PowerWater Kit (Qiagen) following the manufacturer’s protocols. The DNeasy PowerSoil Kit (Qiagen, Carol Stream, IL) was used to extract DNA from sand and sediment samples. DNA concentrations were quantified using the Quant-iT dsDNA Broad-Range Assay Kit (Invitrogen, Carlsbad, CA) on Cytation3 Cell Imaging Multi-Mode Reader (BioTek Instruments, Inc., Winooski, VT).

The presence of human, dog, and gull fecal materials was determined using qPCR, and the presence of horse feces using conventional PCR, with HF183 (Bernhard and Field, 2000; Green et al., 2014) and HumM2 (Shanks et al., 2009) as human-associated fecal markers, DogBact (Dick et al., 2005; Sinigalliano et al., 2010) as the dog marker, Gull2Taqman (Lu et al., 2008; Sinigalliano et al., 2010) as the gull fecal marker, and HoF597 (Dick et al., 2005) as the horse marker. These host-specific markers were selected based on their performances as thoroughly evaluated in a previous study (Boehm et al., 2013). A TaqMan version of the HF183 assay that incorporates an internal amplification control was used as the primary assay to detect human fecal waste (Green et al., 2014). The HumM2 qPCR assay is considered more specific but less sensitive to human feces compared to the HF183 assay (Boehm et al., 2013), and was only performed for HF183 positive samples in this study, and detected in 19.1% of HF183 positive samples (Supplementary Tables 1, 2, 5, 6). The Entero1A marker was used to quantify ENT in units of cell equivalents (c.eq.) per 100 ml for comparison to culture-based measurements by assuming a ribosomal RNA copy number as 6 for *Enterococcus* (Oana et al., 2002; Haugland et al., 2012). The *ttr* gene was used to quantify *Salmonella* spp. bacteria (Malorny et al., 2004). Sample inhibition was assessed using an internal amplification control (IAC) incorporated in the HF183 TaqMan qPCR assay (Green et al., 2014). All qPCR assays were performed using the TaqMan Environmental Master Mix 2.0 (Life Technologies, Grand Island, NY) on a Bio-Rad CFX96 Real-Time PCR Detection System (Hercules, CA). Synthesized plasmid DNA containing qPCR targeted sequences were serially diluted to generate standard curves for all qPCR assays. All samples and standards were analyzed in triplicate with triplicate no-template controls included for each plate. Human adenovirus was quantified using ddPCR with a Bio-Rad QX200 Droplet Digital PCR System (Hercules, CA) as previously published (Steele et al., 2018). The conventional PCR, qPCR and ddPCR procedures are described in the SI Methods. Samples with two or more replicates amplifying within the range of the standard curve were considered to be within the range of quantification (ROQ) and were quantified. Samples with two or more replicates amplifying below the lowest standard were considered detected but not quantifiable (DNQ), and samples with one or zero replicates amplifying were considered not detected (ND), as described previously (Ervin et al., 2014).

### Bather Shedding

For studying bather shedding as a potential source of FMPs, surf zone water samples were collected on high visitation weekends or holidays (Jul 3rd, Aug 12th, and Sep 4th, 2017) during the morning and afternoon. Surf zone waters were also sampled at site G03 at different times of the day (late afternoon, and again in the early morning and mid-afternoon of the following day) on five occasions for evaluating if water defecation was occurring by potential campers overnight (Supplementary Table 6). During bather shedding studies, the counts of people were grouped into people “in the water” including swimmers or anyone recreating in the surf zone (bather), and people “on the sand,” meaning people recreating but not in the water (i.e., walking, sitting on the sand, and exercising).

### Statistical Analyses

Statistical analyses included Wilcoxon tests (Mann-Whitney for two categories, or Kruskal-Wallis with Steel-Dwass pairwise comparisons for three or more categories) and Spearman’s rho rank correlation performed using JMP10 (SAS, Cary, NC). Over or under range values were adjusted prior to statistical analysis to be above the highest, and below the lowest, quantified values, respectively. FIB values were treated as follows, < 10 = 0 (log scale), and > 24,196 = 25,000. For qPCR assay results, DNQ values were set to 1.8 (log scale) and ND values were set to 1.3 (log scale).

## Results

### FMPs in Surf Zone Waters

**FIGURE 1:**
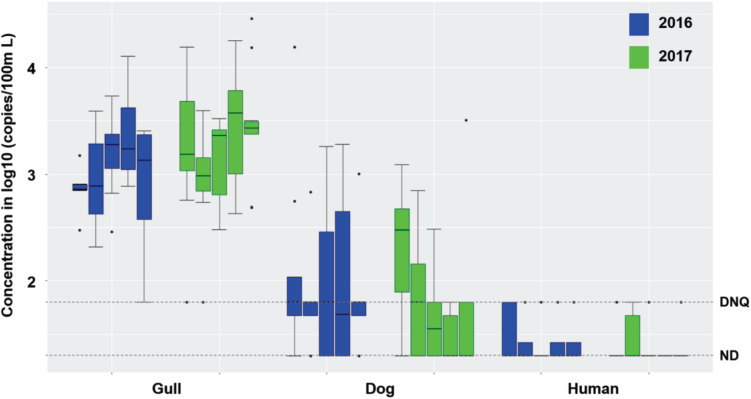
Boxplots of gull, dog and HF183 human fecal marker concentrations in surf zone waters collected from 5 sites (G01-G05, from left to right in each set of boxes) of GB in 2016 (8 samples per site and 40 samples in total) and 2017 (10 samples per site and 50 samples in total) during dry weather (Supplementary Table 1). DNQ (1.8 on log10 scale) and ND (1.3 on log10 scale) levels are shown in the figure as dotted lines.

Surf zone waters were sampled at 5 GB sites in 2016 and 2017 (Supplementary Figure 1). During dry weather (*n* = 90 samples), there were no exceedances of single sample AB411 criteria for TC or ENT, but 17.5 and 18% of the samples exceeded EC criteria in 2016 and 2017, respectively (Supplementary Table 1). Gull markers were detected in all surf zone water samples, with 97.5 and 96% of samples at quantifiable levels in 2016 and 2017, respectively; dog markers were also detected in 67.5 and 52% of water samples (in 2016, and 2017, respectively) at DNQ or quantifiable levels (Figure 1). The human marker HF183 was detected in 25 and 14% of surf zone water samples in 2016 and 2017, respectively, all at DNQ levels (Figure 1). *Salmonella* spp. bacteria were not found in any sample, and human adenovirus was detected in one sample across the 2 years (Supplementary Table 1). In another regional study, the potential for non-specific detection of HF183 was ruled out, since there were no detections when performing the assay using DNA extracted from gull feces (SI Results).

Although the estuarine slough was bermed with sand and thus closed to the surf zone in 2016, it breached and flowed into the surf zone in 2017; still, the breaching did not appear to affect surf zone water quality as FMPs were similar across 2016 and 2017 (Supplementary Table 1 and Figure 1) except for higher concentrations of TC in 2017 (Mann-Whitney, *p* < 0.001, *n* = 90). There was also no significant difference in FIB or host fecal marker concentrations across the 5 surf zone sampling locations across both years (Supplementary Table 1 and Figure 1; Kruskal-Wallis, all *p* > 0.05, *n* = 90), except for ENT by Entero1A (*p* < 0.001). When site to site variations were evaluated within each study year, FMPs did not vary among the 5 surf zone locations in 2016, and only TC and ENT by Entero1A showed significant variance among the 5 locations in 2017 (both *p* < 0.03, *n* = 50). Taken together, these results indicate that the slough breaching did not affect fecal markers in the surf zone. Across the 2016 and 2017 dry weather results, the surf zone concentrations of TC, EC, and ENT by Entero1A were closely correlated (Spearman’s ρ ranging from 0.24 to 0.76, all *p* < 0.03, *n* = 90). Further, the FIB concentrations correlated with gull marker concentrations (ρ ranging from 0.28 to 0.46, all *p* < 0.01, *n* = 90), but not with dog or human marker HF183. Such correlation and the prevalence of gull markers indicated that gulls were the major sources of FIB in the surf zone during dry weather.

For the three rain events occurring during the study periods (*n* = 9 samples), exceedances of single sample AB411 criteria in 2016 were recorded once (17%) for TC and ENT in surf zone waters, and for 33% of EC measurements; there were no FIB criteria exceedances in 2017 (Supplementary Table 1). The correlations among gull markers, ENT, and ENT by Entero1A were stronger for wet weather samples (ρ ranging from 0.75 to 0.89, all *p* < 0.05, *n* = 9), indicating that rain events transported gull fecal deposits into surf zone waters.

### FMPs in Watershed Waters and Sediments

**FIGURE 2:**
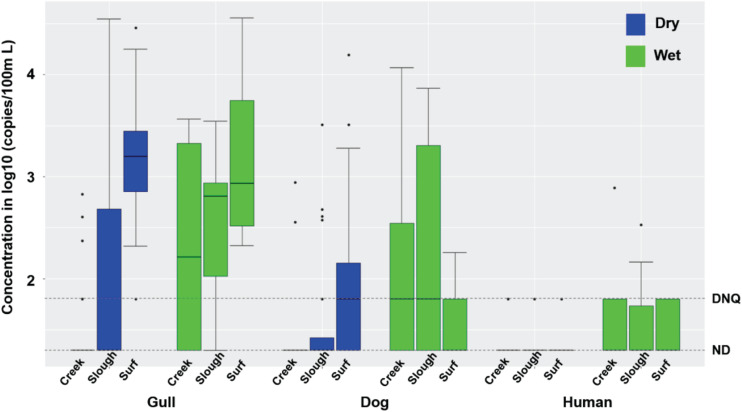
Boxplots of gull, dog and HF183 human fecal marker concentrations in creek, slough, and surf zone (from left to right) water samples collected from watershed and surf zones of GB in 2016 and 2017 during dry and wet weathers (Supplementary Tables 1, 2). The number of creek, slough, and surf zone samples was 40, 40, and 90 during dry wet weather, respectively, and 9, 7, and 9 during wet weather, respectively. DNQ (1.8 on log10 scale) and ND (1.3 on log10 scale) levels were shown in the figure as dotted lines.

Inland waters contained much higher concentrations of FIB under dry weather conditions compared to surf zone waters, with 70 and 75% of TC, 65 and 57.5% of EC, and 22.5 and 30% of ENT exceeding AB411 single sample criteria in 2016 and 2017, respectively (Supplementary Table 2, Mann-Whitney test, all *p* < 0.001, *n* = 80). Gull markers were present in 7.5 and 52.5% of inland water samples in 2016 (40 samples) and 2017 (40 samples), respectively, and dog markers were present in 2.5 and 27.5% water samples in 2016 and 2017, respectively (Figure 2). The concentrations of gull and dog markers were both significantly lower in inland waters than in surf zone waters (Figure 2; Mann-Whitney test, both *p* < 0.0001, *n* = 170). Human marker HF183 was present in 5 and 7.5% of inland water samples in 2016 and 2017, respectively, while *Salmonella* spp. bacteria were not present in any dry weather sample, and human adenovirus was present in only one sample (2.5%) in 2016 and one sample (2.5%) in 2017, respectively (Supplementary Table 2). Horse markers were not detected in water samples from the Atascadero Creek and lower slough sites G07 through G09 (Supplementary Figure 1).

During dry weather, inland waters had significantly higher concentrations of gull and dog markers in 2017 relative to 2016 (Mann-Whitney test, both *p* < 0.05, *n* = 80), while human marker HF183 and FIB did not vary significantly (Supplementary Table 2). FIB concentrations significantly differed across the sampling locations including the slough, and upstream into Atascadero Creek, San Jose Creek, and San Pedro Creek (Kruskal-Wallis, all *p* < 0.002, *n* = 80), with the highest average concentrations in San Jose Creek and San Pedro Creek waters, followed by the slough, and Atascadero Creek. Gull marker concentrations also significantly differed across the slough and the three creeks (Figure 2; Kruskal-Wallis, *p* = 0.038, *n* = 80), with the slough containing the highest average concentrations. Dog and human marker HF183 detections did not significantly differ across the slough and creeks (Figure 2). Although the slough contained significantly higher FIB concentrations than surf zone waters (Supplementary Tables 1, 2, Mann-Whitney test, all *p* < 0.01, *n* = 80), there were significantly fewer gull and dog markers in the slough than surf zone waters (Supplementary Tables 1, 2; Mann-Whitney test, both *p* < 0.01, *n* = 80), and the average human marker HF183 detections appeared lower than surf zone waters although not significantly (Supplementary Tables 1, 2, *p* = 0.2). These results indicated that the slough was not the source of host fecal markers in surf zone waters. Overall, significant correlations were found between the concentrations of TC and EC (Spearman’s ρ = 0.46, *p* < 0.00001, *n* = 80), ENT and ENT by Entero1A (ρ = 0.67, *p* < 0.00001, *n* = 80), and gull and dog markers (ρ = 0.42, *p* = 0.0003, *n* = 80) in inland water samples.

Rain events resulted in increased FMPs in inland waters, with gull markers detected in 60 and 83.3% of samples, dog markers in 70 and 50% of samples, and human marker HF183 in 40 and 50% of samples in 2016 (*n* = 10 samples) and 2017 (*n* = 6 samples), respectively, mostly at quantifiable levels (Figure 2 and Supplementary Table 2). *Salmonella* spp. were present in 40 and 33.3% of water samples in 2016 and 2017, respectively, and human adenovirus was found in 33.3% of water samples in 2017. The concentrations of host fecal markers and *Salmonella* spp. were higher during rain events compared to dry weather conditions (Mann-Whitney test, all *p* < 0.02, *n* = 96), and dog markers, human marker HF183, and *Salmonella* spp. were correlated with each other (Spearman’s ρ ranging from 0.65 to 0.84, *p* < 0.01, *n* = 16). These results indicated that most of the assayed fecal markers, and *Salmonella* spp., were mobilized with fecal deposits into inland waters during runoff-generating rainfall events. The only exceptions were horse markers which were not detected. There was mostly no significant interannual difference for FMPs across 2016 and 2017, except that EC was higher in 2016.

In contrast to sampled waters, watershed sediments contained much lower levels of fecal markers, with no human HF183 or dog markers detected in any sediment sample, and gull markers detected at 2 locations in the slough (G06/G06A and G13 in Supplementary Figure 1 and Supplementary Table 3). FIB were quantifiable in many samples, which is common in freshwater sediments (Pachepsky and Shelton, 2011).

### Studies of Beach Sands and Infrastructure at GB

In intertidal beach sands (Supplementary Figure 1), FIB concentrations were very low or non-detectable, and human HF183 or dog markers were not detected (Supplementary Table 3). Gull markers were present in 6.7% (1 of 15) and 13.3% (2 of 15) of sand samples in 2016 and 2017, respectively, and human adenovirus was found in 1 sand sample in 2016. There were no above background levels of dye detected in groundwater, surf zone, and slough water samples collected after the addition of dye to infrastructure including the bathrooms, RV sewer connections, and sewage lift stations at GB (Supplementary Figures 4–7). Furthermore, no human marker HF183, dog markers, *Salmonella* spp., or human adenovirus were detected in any groundwater samples collected from groundwater wells; gull markers were present in 50% of groundwater samples (Supplementary Table 4).

### Nearshore and Offshore Waters, Sediments, and Treated Wastewater Effluent

**FIGURE 3:**
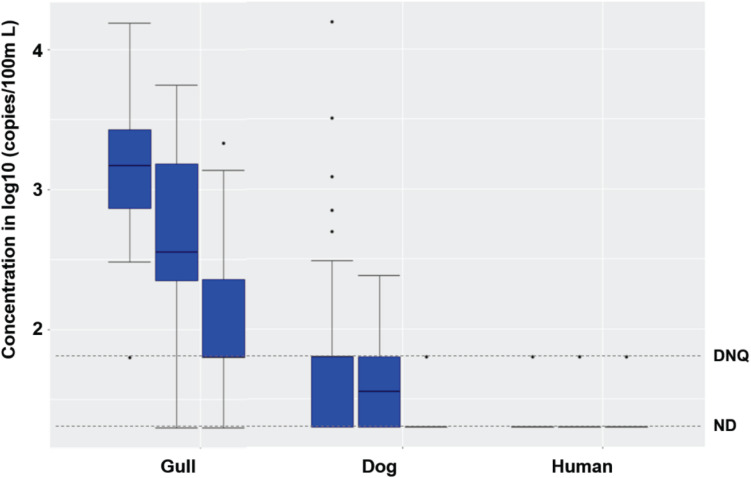
Boxplots of gull, dog and HF183 human fecal marker concentrations in surf zone, nearshore, and offshore (from left to right in each box set, one set per marker) water samples in 2016 and 2017. The number of surf zone, nearshore, and offshore samples was 40, 24, and 32, respectively. DNQ (1.8 on log10 scale) and ND (1.3 on log10 scale) levels were shown in the figure as dotted lines.

Synchronous sampling of surf zone and, from watercraft, nearshore and offshore waters in both 2016 and 2017 (Supplementary Figure 3) revealed decreasing concentrations of FIB from the surf zone to nearshore and further offshore (Supplementary Table 5, Kruskal-Wallis, all *p* < 0.03, *n* = 96). This decreasing spatial trend was also observed for gull markers in both 2016 and 2017 (both *p* < 0.0001), and dog markers in 2017 (*p* < 0.03), but not for human marker HF183 in either 2016 or 2017, possibly due to low detection frequencies (Figure 3).

When combining the 2016 and 2017 data, the FIB, gull and dog fecal marker concentrations decreased spatially from the surf zone to nearshore and further offshore (Figure 3; Kruskal-Wallis, all *p* < 0.01, *n* = 96). Human marker HF183 also decreased in the same spatial pattern, with detection ratios of 10, 8, and 6% in the surf zone, nearshore, and offshore, respectively (Figure 3). The FIB or fecal marker concentrations within the 5 surf zone sampling sites, 3 nearshore sites, or 4 offshore sites were mostly spatially invariant (Kruskal-Wallis, all *p* > 0.05), except for the gull marker within the 5 surf zone sites (*p* = 0.047, *n* = 40). Overall, FIB and ENT by Entero1A concentrations across all surf zone, nearshore, and offshore water samples correlated significantly with each other (Spearman’s ρ ranging from 0.36 to 0.73, *p* < 0.001, *n* = 96), and further with gull markers (ρ ranging from 0.23 to 0.66, *p* < 0.03, *n* = 96). *Salmonella* spp. and human adenovirus were not detected in any surf zone, nearshore, or offshore sample of either 2016 or 2017. No significant interannual variation was observed for any host fecal marker across 2016 and 2017 (Mann-Whitney test, all *p* > 0.05, *n* = 96). Nearshore marine sediments were mostly devoid of fecal markers, except that gull marker was detected in one sample; sediments contained very low to non-detectable FIB (Supplementary Table 3).

For treated effluent collected from a diffuser port on the WWTP outfall and marine water collected over the outfall diffuser at 1, 9, and 18 m depth from the surface (Supplementary Figure 3), the concentrations of EC, ENT, ENT by Entero1A, and dog markers were similar or lower when compared to nearshore and offshore waters, and significantly lower than surf zone waters (Supplementary Table 5, Mann-Whitney test, all *p* < 0.01). The concentrations of gull markers were also significantly lower in waters from and over the outfall diffuser than nearshore, offshore, and surf zone waters (all *p* < 0.001), and human marker HF183 levels were similar among these water samples (all *p* > 0.05) (Figure 3 and Supplementary Table 5). Only one sample with human marker HF183 was detected from the diffuser port on the WWTP outfall, and this was at the DNQ level (Supplementary Table 5). In addition, there were only two detections of HF183 in the water over the outfall diffuser (one DNQ and 131 copies/100 ml, both at 18 m depth). These results indicated that the treated wastewater effluent discharging from the ocean outfall was unlikely to be the major source of chronic human fecal markers detected in surf zone waters. However, given complex nearshore ocean circulation patterns, the influence of the WWTP effluent outfall discharge on surf zone HF183 detections remained uncertain.

### Results of Studies to Determine the Role of Bather Shedding

The possible effects of bather shedding on surf zone water quality were studied by counting bathers in the water at the time of sampling surf zone waters, including in the mornings and afternoons of holidays and high visitation weekends (Supplementary Tables 6, 7) when many people were recreating at the beach and in the water. There was also a sub-study intended to determine if water defecation might be happening overnight in the surf zone by beach campers; this was investigated by comparing HF183 human marker detections in the morning with those in the previous late afternoon (ca. 6–7 pm), and also with the following mid-afternoon (ca. 3–4 pm) (Supplementary Tables 6, 7). This timing was selected expecting that overnight water defecation would lead to higher morning human marker detections; it also allowed for inferring possible marker decay due to sunlight during the day. Besides the sub-studies of holidays and high visitation weekends, and of possible water defecation, the human beach visitor census at the time of surf zone water sampling was recorded during all other morning sampling campaigns for surf zone waters during dry weather in 2017 (Supplementary Table 7). The bather census was not recorded during 2016 surf zone water sampling.

In the studies of surf zone waters during holidays or high visitation weekends, human marker HF183 was detected in 1 of 15 morning samples, and 2 of 15 afternoon samples. Thus, as with FIB concentrations (Supplementary Table 6), the human marker HF183 concentrations did not vary significantly across the mornings and afternoons (Supplementary Table 6, Mann-Whitney test, all *p* > 0.05, *n* = 30). No human marker HF183 was detected in any water sample collected during the water defecation study, and FIB concentrations did not vary across the three different times of day (Kruskal-Wallis, *p* > 0.05, *n* = 15). However, HF183 markers were detected in 7 of 50 other morning surf zone samples during dry weather in 2017 (watershed to surf zone transect, and surf zone to nearshore to offshore transect; Supplementary Table 7) when the human visitor census was recorded at the time of sampling.

Across all surf zone water data for which the human census was recorded at the time of sampling, HF183 concentrations and total counts of people—whether in the water (bather) or on the sand—were uncorrelated (Supplementary Table 7, Spearman’s one tailed ρ < 0.1, both *p* > 0.29, *n* = 93), particularly in the morning (ρ < 0, both *p* > 0.58, *n* = 70). There was no relationship between human marker HF183 detections and the counts of people on the sand in the afternoon (ρ = 0.19, *p* = 0.19, *n* = 23). However, there was a significant correlation between the number of afternoon bathers vs. surf zone water sample HF183 concentrations (ρ = 0.36, *p* = 0.04, *n* = 23). Furthermore, the correlation between HF183 concentrations and bather counts appeared to be stronger when only afternoon samples from holidays and high visitation weekends were considered (ρ = 0.46, *p* = 0.04, *n* = 15), i.e., when there were more people bathing at GB (2.7 bathers in average per sampling location). This was in contrast to morning samples from holidays and high visitation weekends wherein, with only 0.5 bathers in average per location, there was no correlation between HF183 concentrations and bather counts observed (ρ < 0, *p* = 0.71, *n* = 15). These results suggest that people recreating in the water during the afternoon contributed to human fecal markers observed in the surf zone at GB, but leaves open that another source could be responsible for observations of HF183 in morning surf zone water samples.

## Discussion

Animal fecal materials, particularly from gulls and dogs, have been identified as major sources of fecal contamination in recreational beach surf zone waters in previous studies (Wright et al., 2009; Converse et al., 2012; Goodwin et al., 2016) as well as in this study, possibly due to many resident gulls and dog walking at GB. Corresponding management practices such as using falconry or dogs to control gulls can dramatically reduce the levels of FIB in surf zone waters (Converse et al., 2012; Goodwin et al., 2016). However, due to host specificity, pathogens from the animal feces may pose less serious risks to public health than human feces (Schoen and Ashbolt, 2010), although some risk does stem from gull fecal exposure (Brown et al., 2017a). Thus, such FIB control actions, even if successful, may not fully achieve the desired level of health risk reduction for swimmers.

Leaking sewers, storm drains, creeks, and rivers can deliver FMPs of human concern to surf zone waters. Also, although specific sources such as treated wastewater or reclaimed water may be confounded by dead microbial cells or free human marker DNA, sources such as human fecal materials or sewage from leaking sewer lines carry a risk to human health from associated pathogens. Thus, sources of even low levels of human fecal markers should be discerned for better evaluating potential human health risks and for guiding suitable management actions.

In other studies, beach berms breaching at coastal sloughs significantly controlled watershed influences on surf zone water quality (Riedel et al., 2015; Sikich et al., 2018). Here, the slough was bermed in 2016 and breached in 2017 (Supplementary Figure 1). However, no significant variations in human fecal marker HF183 and pathogen levels in surf zone waters under dry weather were observed across 2016 and 2017, or among the 5 sampling locations (Figure 1). This might owe to the lower concentrations of human fecal marker HF183 in the slough compared to the surf zone waters during dry weather. Although rain events can mobilize human and dog fecal materials to watershed waters, thereby increasing levels of human and dog fecal markers as well as pathogens (e.g., *Salmonella* spp.) (Steele et al., 2018), runoff-generating rain events were rare in this study area (twice in 2016 and once in 2017), and the slough was continuously bermed in 2016. In other studies, creek sediments were persistent sources of contamination including human fecal markers to creek waters (Frey et al., 2015; Kim and Wuertz, 2015), but watershed sediments, intertidal sands, and nearshore sediments in this study were all devoid of human and dog markers (Supplementary Table 3). This indicated that the fecal markers mobilized into creek waters during rain events were not accumulating in creek sediments; they were also not stored in beach sands and thereby becoming long-term slow-release sources into surf zone waters. Taken together, all evidence would suggest that human fecal markers detected in the GB surf zone during this study did not originate in the watershed.

Infrastructure such as sewer lines in this study were not leaking. The low concentrations of human fecal marker HF183 in treated effluent discharging from the WWTP offshore ocean outfall (67% ND, 33% DNQ) and measured in marine water above the outfall diffuser at 18 m depth from the surface [60% ND, 20% DNQ, 20% quantifiable levels (131 copies/100 ml)]—with likely further marker dilution, decay, and predation during ocean transport (Carneiro et al., 2018)—did not appear to fully explain GB surf zone fecal contamination. Further, in a parallel study, it was concluded that there were no regional background levels of HF183 human markers or pathogens in surf zone waters, based on the results of sampling the Arroyo Hondo reference beach located up-coast of the Santa Barbara region (SI Results).

The results of this study point to bather shedding as a source of HF183 human fecal markers in surf zone waters, particularly in the afternoon when more bathers were present. Bather shedding has been demonstrated to be a possible source of FIB such as enterococci and human pathogens such as methicillin resistant *Staphylococcus aureus* to surf zone waters (Elmir et al., 2007, 2009; Plano et al., 2011). Correlations between bather density and levels of human waterborne pathogens as well as enterococci in marine recreational beach waters have also been observed in summer weekends previously (Graczyk et al., 2010). However, direct correlations between bathers and human fecal markers have not been reported yet. Although the low levels of human marker HF183 in the surf zone waters (at the DNQ level) of this study were well below public health risk thresholds (Boehm et al., 2015; Brown et al., 2017b), there was a significant correlation between bather counts and human fecal marker HF183 for afternoon samples, especially during holidays and high visitation weekends. The human markers detected in the morning could have originated from bather shedding in the previous afternoon, but it is also possible that the WWTP outfall was influential on human marker detections due to diffuser discharge ocean circulation patterns overnight, which were unstudied. This is worthy of further consideration given that HF183 was detected in 11.4% of all morning samples (8 of 70, Supplementary Table 7), which was higher than for the afternoon samples (2 of 23, or 8.7%), and similar to that for holiday and high visitation weekend afternoon samples (2 of 15, or 13.3%). There were few bathers in the surf zone in the morning (Supplementary Table 7) which may point to sources of HF183 varying at different times.

In another study in the region (Li et al., 2021), bather counts significantly correlated with human fecal marker HF183 in surf zone waters, particularly in the afternoon, while effects of the WWTP outfalls were unknown and could not be ruled out. However, in a prior MST study of another high visitation regional beach (Ervin et al., 2014), low level HF183 markers were chronically detected in the surf zone and there was no nearby WWTP outfall. Although swimmers were not counted in that prior study, the absence of a WWTP outfall at this other high visitation beach with chronic low surf zone HF183 would reinforce—based on this study’s results—that bather shedding might have been responsible. Bather shedding might further explain the low but chronic presence of human fecal markers at many beaches along the California coastline (Santoro and Boehm, 2007; McQuaig et al., 2012; Russell et al., 2013; Riedel et al., 2015; Cao et al., 2017; Jennings et al., 2018). Our results indicate that afternoon sampling should be included and compared with morning sampling, rather than assume that afternoon sunlight-mediated decay confounds human marker detections. Yet, additional research should be performed to further understand relationships between bather shedding and human fecal contamination in recreational surf zone waters, including at different times of the day, such that impacts of bather shedding on microbial water quality and potential associated public health implications can be understood. Regardless, the possibility that multiple, permanent sources—in this case a WWTP outfall, and bathers routinely using the beach—can explain low chronic HF183 surf zone detections suggests that there are likely HF183 thresholds that are the lowest achievable at popular bathing beaches.

## Data Availability Statement

The original contributions presented in the study are included in the article/Supplementary Material, further inquiries can be directed to the corresponding author/s.

## Author Contributions

DL and LV planned and performed research, analyzed data, and co-authored manuscript. BS and JM planned research and co-authored manuscript. JE and ND planned and performed research and co-authored manuscript. PH secured project funding; led and planned the research; co-analyzed the results; co-authored the manuscript. All authors contributed to the article and approved the submitted version.

## Conflict of Interest

BS and JE were employed by the company Geosyntec Consultants. The remaining authors declare that the research was conducted in the absence of any commercial or financial relationships that could be construed as a potential conflict of interest.

## Footnotes

1 http://brc.healthebay.org/
