# Supplementary material for: Bather Shedding as a Source of Human Fecal Markers to a Recreational Beach
Source: Front Microbiol. 2021 Jun 25;12:673190. doi: 10.3389/fmicb.2021.673190 (PMC8269448; doi:10.3389/fmicb.2021.673190)
Supplement: Supplementary file 1 [file Data_Sheet_1.pdf]

SUPPLEMENTAL INFORMATION

Bather Shedding as a Source of Human Fecal Markers to a Recreational Beach

Dong Li[1], Laurie C. Van De Werfhorst[1], Brandon Steets[2], Jared Ervin[2], Jill L. S. Murray[3], Naresh Devarajan[1], Patricia A. Holden[1]*

[1]Bren School of Environmental Science & Management, University of California, Santa Barbara

[2]Geosyntec Consultants, Santa Barbara, CA 93101

[3]Creeks Division, Department of Parks & Recreation, City of Santa Barbara, CA 93102

*Corresponding author:

Patricia A. Holden

E-mail: holden@bren.ucsb.edu

Supplemental Methods

Dye studies

To determine whether sanitary infrastructure including sanitary sewer lines, laterals, and pressurized force mains were leaking and then impacting groundwater or surface waters through high velocity groundwater or preferential flow pathways, dye studies were performed in 2016 by flushing a non-toxic fluorescent dye (2.5% Rhodamine WT dye, Cole-Parmer, Vernon Hills) down toilets in the five bathrooms on GB and adding directly to the three recreational vehicle (RV) sewer connections and two sewage lift stations located at GB (Fig. S4). Six groundwater, four surf zone, and three GB slough sampling locations were chosen at GB for sampling during the dye tests (Fig. S4). Sampling locations were selected nearby to allow for monitoring areas where the dye might travel if it were to reach surface waters from leaking infrastructure.

Six temporary groundwater monitoring wells were installed on 10/10/16 between the west end of GB and just east of the GB Pier (Fig. S4). To install each well, dry surface sand was first removed from the area using a shovel. A 3.25-inch hand auger (AMS, Inc., American Falls, ID) was then used to core down to groundwater. A PVC sampling pipe consisting of a 5 ft screened section of 1 inch PVC with a threaded cap on one end and a 5 ft section of 1 inch PVC casing on the other end (Environmental Service Products, Irvine, CA) was then placed in the auger hole and pushed so that the screened section of PVC was approximately 1 ft below the surface of the groundwater table. In shallow wells (<4 ft to groundwater), only the PVC pipe with a screened section was used. Sand was packed around the sampling pipe, and the pipe was then cut off above the sand surface. One quarter (0.25)-inch diameter polypropylene tubing (Cole-Parmer, Vernon Hills, IL) was then pushed into the bottom of the sampling pipe and cut off at the top. A 1-inch PVC slip cap was used to close the top of the sampling pipe between sampling events. The section of PVC protruding from the sand was painted orange to increase visibility and labeled to prevent tampering. The depth to groundwater was between 1 ft and 6 ft below ground surface for all wells installed at GB.

Before the fluorescent dye was added, background samples were collected from each of the surface and groundwater sampling locations. Five rounds of background sampling were conducted for all groundwater, slough, and surf zone locations over a two-day period (10/10/2016-10/11/2016). To evaluate the bathrooms and associated sanitary sewer lines, one gallon of fluorescent dye was added to each of the 5 bathrooms on GB. One gallon of dye was also divided

between the three RV sewer connections located at the Ranger Station (near LS2 and BR5, Fig. S4). Finally, one gallon of dye was added to each of the two sewage lift stations to directly evaluate these structures and the associated force mains. To reduce the load of dye such that the local wastewater treatment plant (WWTP) did not receive a large pulse, the dye addition was performed over two days. On day one (10/12/2016 between 7:00am and 7:45am), dye was added to the three public bathrooms (BR1, BR2 & BR4) and the restaurant (BR3) and on day two (10/13/2016 between 7:40am and 8:15am), dye was added to the ranger station bathroom (BR5), RV connections, and the lift stations (LS1 & LS2) (Fig. S4).

The date and general time of each sample set collected are summarized in Table S8. When high tides did not allow sampling at certain locations, samples were either skipped or collected at a later time. Samples were collected in 50mL centrifuge tubes (Cole-Parmer, Vernon Hills, IL) and immediately placed on ice in the dark. Surface water samples were collected by submerging the collection tube. In the surf zone, samples were collected from between ankle and knee depth. Groundwater samples were collected by pumping water to the surface from wells using a Geotech Geopump 2 Peristaltic Pump (Geotech Environmental Equipment, Inc., Denver, CO). At each well, 3 ft of silicon tubing (Cole-Parmer, Vernon Hills, IL) was connected to the tubing in the well and threaded through the pump head. Groundwater was pumped for at least one minute to clear water from the tubing and well before sampling was performed. Water was then pumped directly into the sampling bottles. One sample from each groundwater well (GW1 through GW6) was also captured and analyzed for FMPs.

All water samples were analyzed in the lab using a Cytation3 Cell Imaging Multi-Mode Reader (BioTek Instruments, Inc., Winooski, VT). The excitation wavelength was 546 nm and the emission wavelength was 590 nm as suggested (Wilson et al., 1986). Standards of 1, 10, and 100 ppb dye in Nanopure water (Barnstead, Thermo Scientific) were used to create the standard curve. Matrix effects were evaluated for all water samples by spiking standards into background samples. The limit of detection in each type of water was defined as the mean plus three times the standard deviation of the background measurements. The maximum background concentration for each water matrix was then defined as twice the limit of detection, and any sample below it was not considered to be a dye detection.

Sample collection and physicochemical analyses

Surf zone water samples were collected in ankle to knee deep water into sterile polypropylene bottles (4 L). Waters from creeks and the slough were collected from the surface by dipping a sterile beaker into the waterbody and then dispensing into the sample bottle. Subsurface marine water was collected by boat using a 2 or 5 L Van Dorn bottle, which was rinsed at least 3 times with sterile Nanopure water prior to sample collection. The Van Dorn bottle was lowered at each site with both ends open. When reaching the sampling depth, the bottle was moved side to side to flush out the interior before the ends were snapped closed. Final effluent samples from wastewater effluent outfall diffuser ports were also collected similarly using the Van Dorn bottles by scientific divers. The divers descended with both ends of the Van Dorn bottle open, and flushed out the interior of the bottle before holding up to a diffuser port. The bottle was held to the port for at least 30 s before snapping the ends closed to capture the effluent sample. Water samples were passed through sterile 25 µm pore size Miracloth (Calbiochem, San Diego, CA) to remove large debris, and stored on wet ice until processing (within 6 h).

For watershed sediments, intertidal sands, and nearshore sediments, approximately 250 g of each composite wet sediment or sand sample was collected by coring and combining sediments or sands from five individual locations at each sampling site. Each core was collected by using a sterile 50 ml polypropylene tube (BD Biosciences, Bedford, MA) and scraping along the surface (1-7 cm depth). Core samples were then combined and homogenized in clean Ziplock plastic bags and stored on wet ice until processing (within 6 hours). Nearshore sediments were collected by scientific divers using modified sterile 60 mL syringes with rubber stoppers. Five cores were collected at each sampling site including a center location followed by locations 3-4 m to the north, east, south, and west directions. At each location, the top 2 cm was brushed away before the barrel of the syringe was uncapped and pushed into the sediment until approximately 3/4 full, at which point the stopper was replaced on the syringe and the plunger gently pushed to expel any water. Sample cores were then stored in clean Ziplock bags and stored on wet ice until processing in the lab. Particle grain sizes of sediment or sand samples were measured with a CILAS 1190 Laser-particle size analyzer (CILAS, Madison, WI) after dispersing 8-10 g of sediment or sand in 30 mL of deionized water for 5 min. Moisture and total organic content were estimated by measuring the loss of weight of approximately 20 g of sample on ignition at 60 ºC and 500 ºC, respectively in a muffle furnace (IsotempTM, Fisher Scientific, USA).

Arroyo Hondo (34.473333 latitude, -120.141111 longitude) was the rural reference beach to the west of Santa Barbara. Dry weather sampling occurred on Jun 21, Aug 19, and Sep 16, 2016 (Li et al., submitted). On each date, water was collected from five surf zone and one creek sampling sites, and intertidal sands were collected from three surf zone locations.

The host specificity of the HF183 human fecal marker was evaluated using fresh gull and bird feces collected from the study beaches. As described previously (Li et al., submitted), fresh gull feces and bird feces were collected from the study beaches by bird baiting onto a clean (new) tarp and scraping the fresh feces into a sterile sampling container (Sterileware Samplit Scoop and Container System, Bel-Art SP Scienceware, Wayne, NJ).

FIB measurement

Total coliform (TC), *E. coli* (EC) and enterococci (ENT) were quantified using the IDEXX Quanti-Tray/2000 method, following the manufacturer's protocols (IDEXX Laboratories, Westbrook, MA). Aliquots from each water sample after passing through Miracloth were used for FIB measurement and appropriate tenfold dilution using sterile Nanopure water was performed prior to FIB analysis. Sample duplicates were performed for at least every 10 samples processed. A method blank using sterile Nanopure water was included for each batch of reagents. For sand or sediment, 5 g was suspended in 25 mL of sterile Nanopure water or 0.2 % hexametaphosphate (Sigma Aldrich, USA), respectively, shaken for 5 min to dislodge microorganisms and settled for 1 min. The supernatant was collected and analyzed using the same protocol for water samples. The FIB enumeration was performed after incubation for 24 h at 35 ºC for TC and EC and 41 ºC for ENT.

DNA extraction, PCR, qPCR and ddPCR

Water samples (up to 2 L) were vacuum filtered through 0.22 µm filters (MicroFunnel Filter Funnels, PALL Co.) until the point of refusal. The volume of water filtered was recorded. For each sampling event, a filter blank was included by filtering approximately 1.5 L of sterile Nanopure water. Filters were stored at -20 ºC until DNA extraction using the DNeasy PowerWater Kit (Qiagen, Carol Stream, IL) following the manufacturer's protocol. For pathogen analysis including *Salmonella* spp. bacteria and human adenovirus, water samples (up to 2 L) were vacuum filtered through 0.45 µm HAWP filters (EMD Millipore, Billerica, MA) until the point of refusal. Filters

were stored at -80 °C until combined DNA and RNA extraction using the RNeasy PowerWater Kit (Qiagen) and following the manufacturer's protocol except for omitting DNase to allow the elution of DNA plus RNA. The DNeasy PowerSoil Kit (Qiagen, Carol Stream, IL) was used to extract DNA from sand and sediments. Extractions were performed in duplicate (0.25-0.5 g wet) for each sample and combined onto a single spin filter prior to the washing and elution steps in the kit. For all (water, sand, and sediment) samples, an extraction blank without any filter was included in each batch of extractions.

Samples from Atascadero Creek and the slough downstream (sites G07 through G09) were analyzed for the HoF597 horse fecal marker (Dick et al., 2005) using conventional PCR as described previously (Ervin et al., 2014). Samples were analyzed in triplicate, and triplicate positive (DNA extracted from horse feces) and negative (DNA-free water) controls were included in each run. PCR products were visualized on 2.2% agarose FlashGel™ DNA Cassettes using the FlashGel™ System (Lonza, Switzerland). Sample replicates with a visible band present at the same size (bp) as the positive control were considered positive. Samples with two or more positive replicates were deemed positive for the HoF597 marker.

Sample inhibition during qPCR was assessed using an internal amplification control (IAC) performed in duplex with the HF183 assay as described elsewhere (Green et al., 2014). In brief, a fixed amount of the IAC was spiked into the reaction mixture (25 copies/reaction), and duplex calibration curves were created. For each HF183IAC plate, the IAC range of quantification (ROQ) was defined as where there was less than a ± 0.75 quantification cycle (Cq) shift from the IAC mean at 10 copies. Next, the inhibition threshold (average IAC Cq values for all standards + 4 SD) and competition threshold (Cq value where the upper bound of the IAC ROQ intersects the HF183 standard curve) were created. A sample was considered inhibited when its IAC Cq was greater than the inhibition threshold and less than the competition threshold. If this occurred, the sample was diluted and re-analyzed. Any plate with amplification of a no-template control replicate was discarded and the samples were re-analyzed. Filter and extraction blanks were incorporated to assess contamination during sample filtration and DNA extraction, respectively. If any filter or extraction blanks amplified, the corresponding samples were flagged and not used in further analysis. For each sampling year, pooled standard curves for each assay were created. A regression analysis was performed on the pooled standard curve and outliers were removed based on standardized residual values of >+3 or <-3. The Lower Limit of Quantification (LLOQ) for each

assay was calculated by taking the average Cq value of the non-outlier standard replicates at the lowest concentration included in the standard curve. Samples with at least two replicates amplifying within the range of the standard curve were considered to be within the ROQ and were quantified. Samples with replicates amplifying below the LLOQ were considered detected but not quantifiable (DNQ), and samples with one or zero replicates amplifying were considered not detected (ND) (Sinigalliano et al., 2013). A summary of the pooled standard curve parameters for each assay and sampling year of this project is provided in Table S9.

Human adenovirus quantification was performed using droplet digital PCR (ddPCR) with a Bio-Rad QX200 Droplet Digital PCR System (Hercules, CA) (Steele et al., 2018). The reaction mixture was made for droplet generation using the Droplet Generator with droplet generation oil. Generated droplets were PCR amplified, including three positive control from ATCC (VR-930D, Human Adenovirus 41) and five negative (no template) control per 96-well plate. Fluorescence measurement was performed with the QX200 Droplet Reader and analyzed using the QuantaSoft software. The fluorescence threshold was manually set at approximately one standard deviation (500-700 fluorescence units) above the negative control signal. All samples were analyzed in duplicate. A total of ≥20,000 droplets for two reactions were generated per sample. The average upper limit of quantification was 10^5 gene copies per 100 mL. For the plate to be included in the analysis, all negative (no template) control reactions were required to have no positive droplets on the plate. To consider a sample positive and included in further analysis, each sample was required to have a minimum of three positive droplets. If a sample had one positive replicate and one negative replicate, a third replicate was analyzed. Samples with two or more positive replicates were considered positive and averaged; samples with one or no replicates amplifying were considered ND.

Supplemental Results

No samples in this study exhibited inhibition during qPCR as determined by the IAC in the HF183 assay. No human HF183 or dog-associated markers or pathogens including *Salmonella* spp. and human adenovirus were detected in any surf zone, lower watershed water, or beach sand sample of the Arroyo Hondo reference beach located up-coast of the Santa Barbara region, indicating that there were no regional background HF183 markers or pathogens in the surf zone. Meanwhile, no

HF183 markers, dog markers, or human adenovirus, were detected in fresh gull and unidentified seabird feces collected from the Santa Barbara area (Li et al., submitted).

References

Dick, L.K., Bernhard, A.E., Brodeur, T.J., Santo Domingo, J.W., Simpson, J.M., Walters, S.P., Field, K.G. (2005). Host distributions of uncultivated fecal bacteroidales bacteria reveal genetic markers for fecal source identification. *Appl. Environ. Microbiol.* 71, 3184−3191.

Ervin, J.S., Van De Werfhorst, L.C., Murray, J.L., Holden, P.A. (2014). Microbial source tracking in a coastal California watershed reveals canines as controllable sources of fecal contamination. *Environ. Sci. Technol.* 48, 9043-9052.

Green, H.C., Haugland, R.A., Varma, M., Millen, H.T., Borchardt, M.A., Field, K.G., Walters, W.A., Knight, R., Sivaganesan, M., Kelty, C.A., Shanks, O.C. (2014). Improved HF183 quantitative real-time PCR assay for characterization of human fecal pollution in ambient surface water samples. *Appl. Environ. Microb.* 80, 3086-3094.

Li, D., Van De Werfhorst, L.C., Steets, B., Ervin, J., Murray, J.L.S., Blackwell, A., Devarajan, N., Holden, P.A. (2021). Sources of low level human fecal markers in recreational waters of two Santa Barbara, CA beaches: roles of WWTP outfalls and swimmers. *Water Res.* (in press)

Sinigalliano, C.D., Ervin, J.S., Van De Werfhorst, L.C., Badgley, B.D., Balleste, E., Bartkowiak, J., Boehm, A.B., Byappanahalli, M., Goodwin, K.D., Gourmelon, M., Griffith, J., Holden, P.A., Jay, J., Layton, B., Lee, C., Lee, J., Meijer, W.G., Noble, R., Raith, M., Ryu, H., Sadowsky, M.J., Schriewer, A., Wang, D., Wanless, D., Whitman, R., Wuertz, S., Domingo, J.W.S. (2013). Multi-laboratory evaluations of the performance of *Catellicoccus marimammalium* PCR assays developed to target gull fecal sources. *Water Res.* 47, 6883-6896.

Steele, J.A., Blackwood, A.D., Griffith, J.F., Noble, R.T., Schiff, K.C. (2018). Quantification of pathogens and markers of fecal contamination during storm events along popular surfing beaches in San Diego, California. *Water Res*. 136, 137-149.

Wilson, J.F., Cobb, E.D., Kilpatrick, F.A. (1986). Fluorometric procedures for dye tracing. 03-A12. USGS: Washington, D.C.

List of Supplemental Tables

Table S1. Sampling details and results of surf zone water in 2016 and 2017.

Table S2. Sampling details and results of watershed waters in 2016 and 2017.

Table S3. Sampling details and results of watershed and nearshore sediments as well as intertidal beach sands at GB.

Table S4. Sampling details and results of groundwater.

Table S5. Synchronous sampling details and results of surf zone, nearshore and offshore in 2016 and 2017, as well as water collected from a port on the treated wastewater ocean outfall diffuser and from marine water over the diffuser at different depths

Table S6. Sampling details and results of surf zone water samples for bather shedding study.

Table S7. Counts of people in water and on sand as well as HF183 human marker concentrations for surf zone samples in 2017.

Table S8. Dye study sample collection and dye addition date and time.

Table S9. qPCR standard curve summary.

List of Supplemental Figures

Fig. S1 Surf zone and watershed water, intertidal sand and watershed sediment sampling locations.

Fig. S2 Map of GB showing beach (between white lines) and sanitary sewer and storm drain infrastructure.

Fig. S3 Sampling locations of surf zone, nearshore, and offshore during synchronized sampling events.

Fig. S4 Groundwater, surf zone, and slough water sampling locations at GB during dye study.

Fig. S5 Groundwater results after dye addition at GB.

Fig. S6 Surf zone results after dye addition at GB.

Fig. S7 Slough results after dye addition at GB.

Table S8. Dye study sample collection and dye addition date and time

Description	Date	Time
Background sample collection	10/10/16	2pm, 8pm
	10/11/16	8am, 2pm, 8pm
Dye addition	10/12/16	7am
	10/13/16	7am
Groundwater and surface water sample collection	10/12/16	8am, 2pm, 8pm
	10/13/16	8am, 2pm, 8pm
	10/14/16	8am, 8pm
	10/15/16 – 10/27/16	Daily, based on tides

Table S9. qPCR standard curve summary.

Summary of the pooled standard curves for each sample year and assay. ROQ = range of quantification. LLOQ = lower limit of quantification. C_q = quantification cycle

Assay	Year	Slope	y-intercept	R^2	Amplification Efficiency[a]	ROQ copies/rxn	LLOQ C_q
HF183IAC	2016	-3.45	38.1	0.997	0.948	10^1-10^6	34.72
	2017	-3.44	38.4	0.998	0.951	10^1-10^5	34.79
HumM2	2016	-3.44	39.3	0.996	0.954	10^1-10^5	35.91
	2017	-3.49	39.0	0.996	0.935	10^1-10^5	35.47
DogBact	2016	-3.41	39.4	0.990	0.964	10^1-10^5	35.89
	2017	-3.54	39.8	0.995	0.915	10^1-10^5	36.21
Gull2TaqMan	2016	-3.46	40.3	0.994	0.945	10^1-10^6	36.83
	2017	-3.40	40.4	0.995	0.969	10^1-10^5	36.98
Salmonella	2016	-3.33	35.2	0.994	0.996	10^0-10^4	35.20
	2017	-3.31	35.0	0.993	1.003	10^0-10^4	35.01
Entero1A	2016	-3.38	38.4	0.997	0.975	10^1-10^6	34.97
	2017	-3.35	37.8	0.994	0.987	10^1-10^5	34.43

[a] Efficiency = $10^{(-1/\text{slope})}-1$

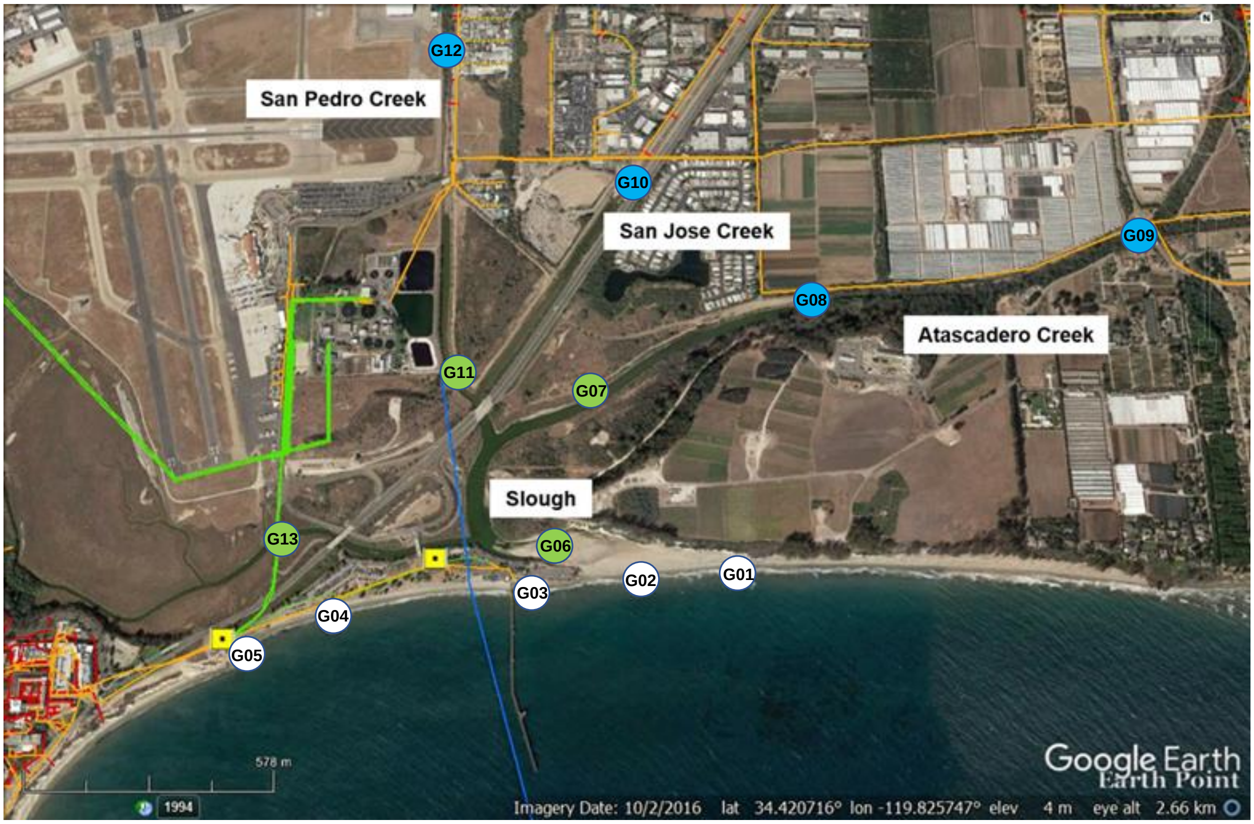

Figure S1. Surf zone and watershed water, intertidal sand and watershed sediment sampling locations. Numbers indicate sampling site. Green circles indicate tidally impacted locations (slough), and blue circles indicate fresh water (creeks). Locations of G06, G10, and G12 varied slightly across sampling years and times, depending on the location of surface water for sampling during the dry summer season (G06A, G10A, G12A, G12B). Surf zone and watershed water samples were collected from sites G01-G13 on 5 dates in 2016 (6/7/16, 7/6/16, 8/3/16, 9/1/16, and 10/4/16) and 5 dates in 2017 (6/13/17, 7/11/17, 8/9/17, 9/7/17, and 9/21/17) during dry weather. During wet weather, surf zone and watershed water samples were collected from sites G02, G03, G04, G06, G08, G10, G12, and G13 on 10/17/16 and 10/28/16 in 2016, and from sites G02, G03, G04, G06, G08, G10, G11, G12, and G13 on 5/7/17 in 2017. Intertidal sands were collected from sites G01-G05 on 3 dates in 2016 (5/26/16, 7/14/16, and 9/8/16) and 3 dates in 2017 (6/13/17, 7/11/17, and 8/9/17). Watershed sediments were collected from sites G06, G08, G11, G12, and G13 on 3 dates in 2016 (5/24/16, 7/12/16, and 9/6/16), and from sites G06, G11, G12, G13 on 3 dates in 2017 (6/13/17, 7/11/17, and 8/9/17).

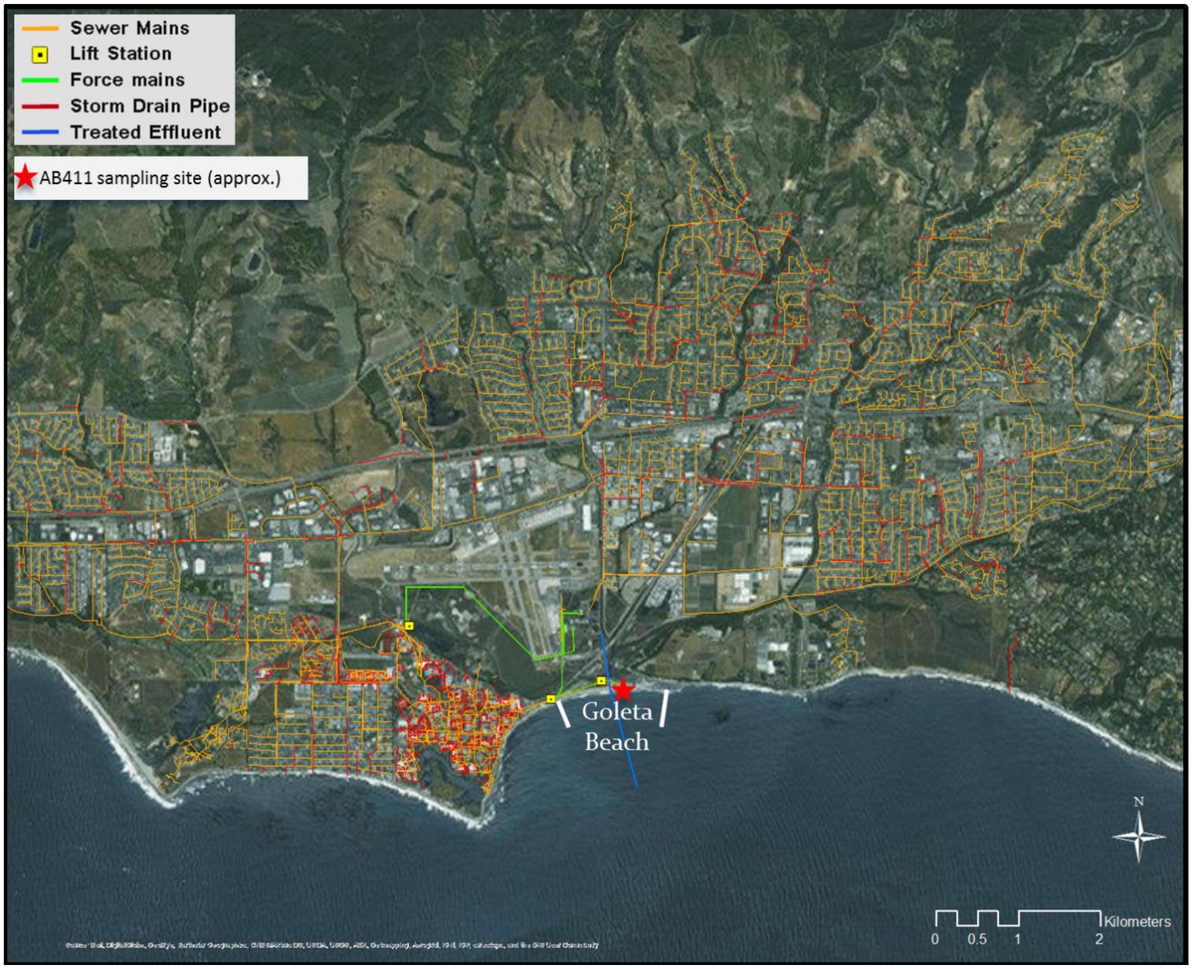

Figure S2. Map of GB showing beach (between white lines that are drawn perpendicular to the shoreline) and sanitary sewer (orange and green lines) and storm drain (red lines) infrastructure. The site at the beach where SB County monitors FIB weekly is marked with a star. The blue line extending away from shore is the location of the Goleta Sanitary District WWTP treated effluent ocean outfall, which extends 5800’ from the shore and terminates with a diffuser section of 36 four-inch diameter ports at a depth of approximately 92’.

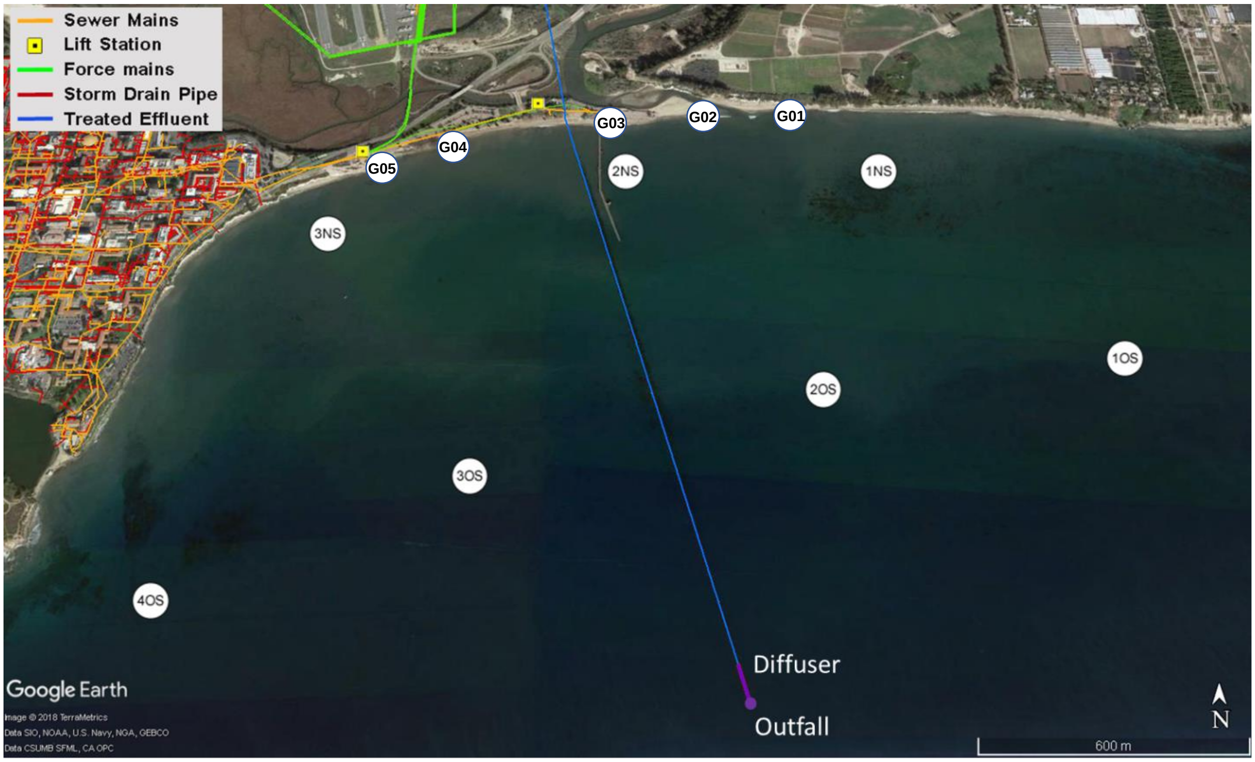

Figure S3. Sampling locations of surf zone (sites G01-G05), nearshore (sites G-1NS through G-3NS), and offshore (sites G-1OS through G-4OS) during synchronized sampling events on 3 dates in 2016 (6/14/16, 8/9/16, and 9/20/16) and 5 dates in 2017 (6/27/17, 7/20/17, 8/22/17, 9/14/17, and 10/10/17). Water samples from a port of the ocean outfall diffuser of Goleta Sanitary District WWTP (purple line) were collected on three dates (8/22/17, 9/14/17, and 10/10/17), and from over the diffuser section at 3 depths (1m, 9m, and 18m from the surface) on 5 dates in 2017 (6/27/2017, 7/20/17, 8/22/17, 9/14/17, and 10/10/17). Nearshore sediments (sites G-1NS through G-3NS) were collected on 6/30/16 and 6/27/17.

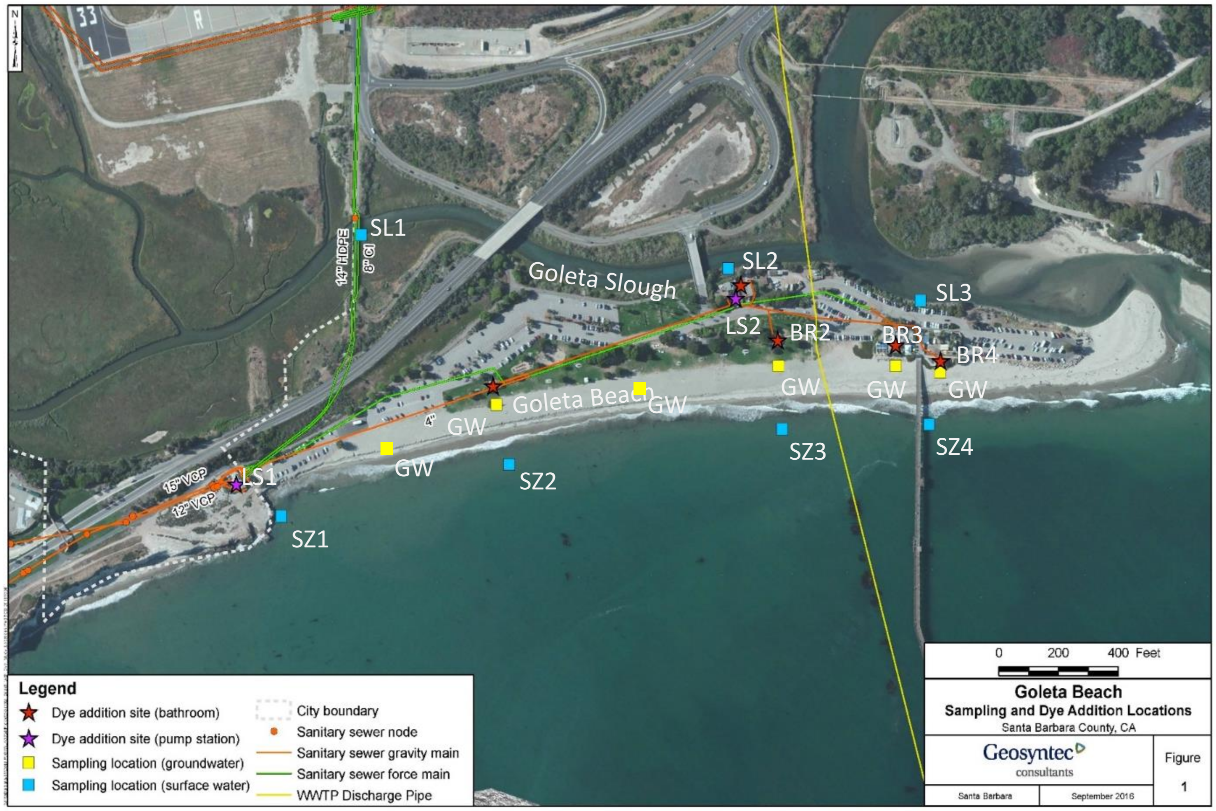

Figure S4. Groundwater, surf zone, and slough water sampling locations at GB during the dye studies. Groundwater wells are symbolized by GW in the figure. SZ1-SZ4 represent the 4 surf zone water sampling locations. SL1-SL3 represent the 3 slough water sampling locations. Line colors are as per the legend.

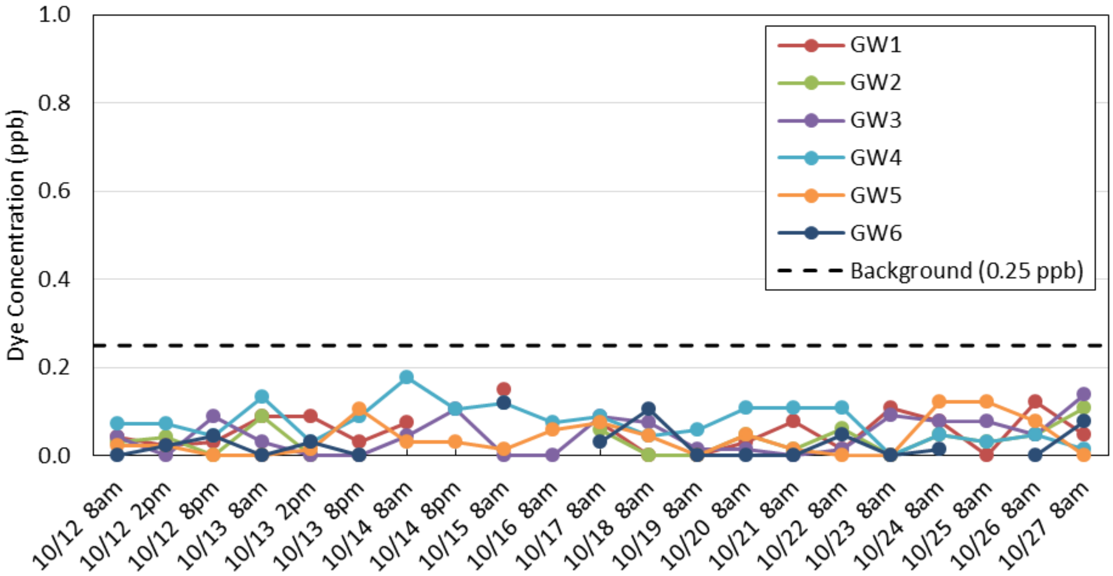

Figure S5. Groundwater results after dye addition at GB. Dye was added between 7:00am and 7:45am on 10/12/2016 and between 7:40am and 8:15am on 10/13/2016.

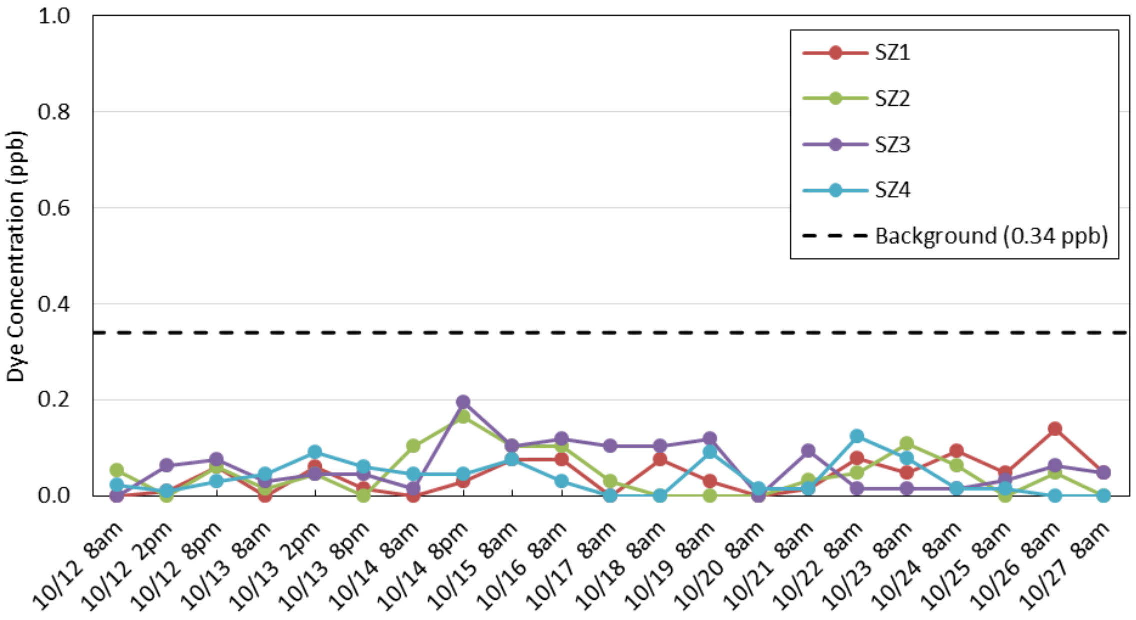

Figure S6. Surf zone results after dye addition at GB. Dye was added between 7:00am and 7:45am on 10/12/2016 and between 7:40am and 8:15am on 10/13/2016.

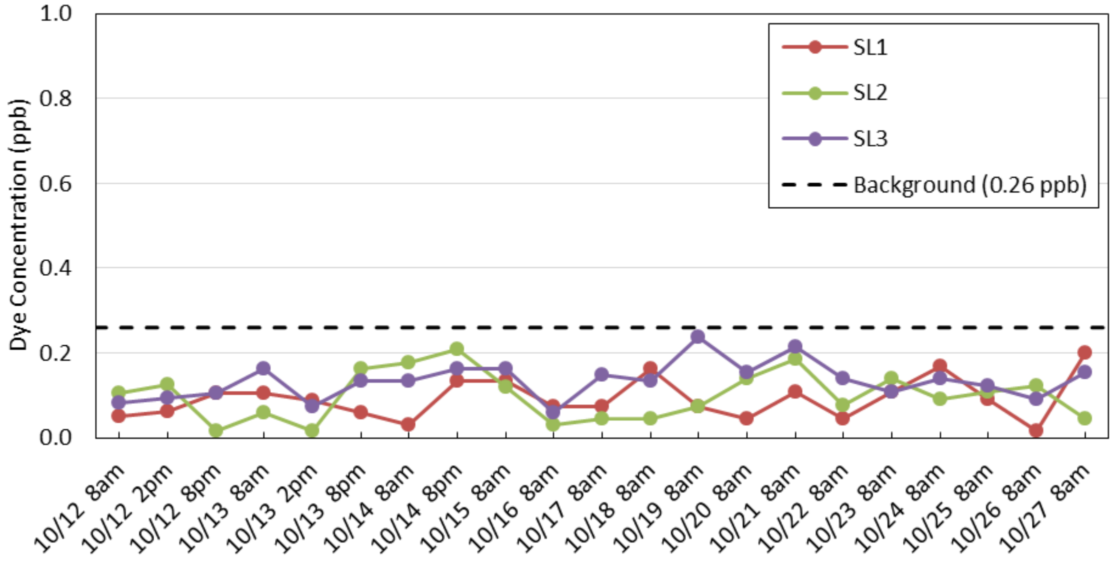

Figure S7. Slough dye study results after dye addition at GB. Dye was added between 7:00am and 7:45am on 10/12/2016 and between 7:40am and 8:15am on 10/13/2016.
